# Papillomavirus DNA not detected in canine lobular orbital adenoma and normal conjunctival tissue

**DOI:** 10.1186/s12917-019-1971-0

**Published:** 2019-07-05

**Authors:** Elizabeth A. F. Schaefer, Shirley Chu, Jacqueline W. Pearce, Jeffrey N. Bryan, Brian K. Flesner

**Affiliations:** 1 Department of Veterinary Medicine and Surgery, University of Missouri College of Veterinary Medicine, 900 East Campus Drive, Columbia, MO 65211 USA; 2 Present address: VCA Canada Vancouver Animal Emergency and Referral Centre, 2303 Alberta St, Vancouver, BC V5Y 4A7 Canada

**Keywords:** Canine lobular orbital adenoma, Papillomavirus, Polymerase chain reaction, Dog, Eye

## Abstract

**Background:**

Canine lobular orbital adenomas are benign tumors that arise from orbital glandular tissue and extend into the orbit, conjunctiva, and third eyelid. Surgical excision is challenging and recurrence rates are high following excision alone. Enucleation and exenteration reduces the likelihood of recurrence, but is a radical therapeutic option for an otherwise visual and comfortable eye. Human papillomavirus causes 4.5% of worldwide cancers in people and has been identified in up to 23% of benign salivary gland tumors. To date, the etiology of canine lobular orbital adenomas has not been established and it is reasonable to consider canine papillomaviruses as an associative agent with benign glandular tumors in dogs. Identification of the underlying etiology of these tumors may help establish treatment or preventative measures. The purpose of this study was to evaluate conjunctival and orbital tissue of phenotypically normal dogs and tissue from canine lobular orbital adenomas for the presence of papillomavirus DNA.

**Results:**

Thirty seven canine lobular orbital adenoma samples (36 formalin fixed paraffin embedded (FFPE) tissue samples from 33 dogs and one freshly collected sample) were evaluated via polymerase chain reaction for the presence of papillomavirus DNA. Conjunctival tissue samples, from 10 dogs with normal ocular examinations, excised immediately following euthanasia, were used as phenotypically normal controls. Three FFPE and one freshly collected tissue samples previously confirmed to be positive for papillomavirus DNA were used as positive controls. PCR products verified positive controls. Papillomavirus DNA was not detected in fresh conjunctival tissue of the phenotypically normal control dogs or in samples of fresh or FFPE canine lobular orbital adenoma tissue.

**Conclusions:**

An association between papillomavirus and the development of canine lobular orbital adenomas is unlikely. Further research is needed to evaluate if other viruses play a role in the pathogenesis of canine lobular orbital adenomas.

## Background

Canine lobular orbital adenomas are benign tumors of epithelial origin and can affect the conjunctiva, eyelids, or third eyelid and extend into the orbit. They occur in middle age to older dogs and have been reported in various breeds [1]. These masses originate from glandular tissue of suspected lacrimal or zygomatic salivary tissue [1, 2]. They may be nodular or solid and are typically lobulated and friable, extending into the orbit. Histopathologic evaluation of these masses by Headrick et al. showed lobulated and well-differentiated tissue of glandular origin arising from either the lacrimal or zygomatic salivary gland. The neoplastic tissue lacks ductular structures and the secretory pattern is mucoserous in nature [1]. They can occur bilaterally and tend to be locally aggressive and frequently recur following surgical excision [1].

Affected dogs are typically visual with no abnormalities of the globe itself, but local disease can result in complications due to orbital mass occupation. Complete excision of these masses is difficult, and radical procedures such as exenteration to obtain clean margins of excision are not ideal as they would result in blindness [1]. Further investigation into microbial, viral, and genetic triggers of these tumors is warranted. A better understanding of the pathogenesis of canine lobular orbital adenoma could aid in both tumor prevention and the development of less invasive, more effective therapies.

Papillomaviruses are non-enveloped, double-stranded DNA viruses of the *Papillomaviridae* family that infect epithelial cells. Human papillomavirus (HPV) is estimated to cause 4.5% of worldwide cancers and is associated with development of epithelial neoplasms in the cervix, anus, penis, and head and neck [3–6]. Additionally, HPV has been identified in salivary gland tumors [7, 8] as well as normal glandular tissue [9], however the role of HPV in the etiology of these tumors has not been determined as other studies have failed to identify HPV in human salivary gland tumors [9–11]. Papillomaviruses contain a single molecule of circular DNA which encodes several early genes involved in tumorigenesis, including, E5, E6, and E7. Together, these genes drive cellular transformation ultimately resulting in malignant conversion. Specifically, E6 targets p53 for degradation via the ubiquitin pathway; E7 binds to the retinoblastoma protein, inactivating it and resulting in downregulation of tumor suppression and promotion of cellular proliferation [4, 12–14]. Additionally, E5 activates or blocks multiple signaling pathways and works in cooperation with the other early gene oncoproteins to result in cell proliferation, protection from apoptosis, and immune evasion [15–19]. While the pathogenesis of high risk HPV in human malignancies has been established, the involvement of E5, E6, and E7 proteins and the exact mechanism for tumorigenesis in dogs has yet to be fully determined. However, Wang et al. illustrate that canine papillomavirus type 2 uses an alternative domain to degrade the retinoblastoma protein and thus E7, of canine papillomavirus type 2, is likely a component of cellular transformation in dogs [13].

In addition, papillomavirus DNA encodes two late genes, including the highly conserved L1 capsid protein gene which is often a target of PCR amplification for viral genome detection [20]. Papillomaviruses have been detected in several epithelial tumors of animals: plaques, papillomas, and squamous cell carcinomas of the skin, cornea, and mucosa of the oral cavity and conjunctiva in dogs, [21–28] equine sarcoids, [29] and cutaneous squamous cell carcinomas and sarcoids in cats [30]. Vaccination with recombinant adenovirus vaccines expressing the E1 and E2 genes of canine papillomavirus resulted in protection from mucosal site warts induced by papillomavirus in beagles [31]. Therefore, if papillomaviruses are identified as causative agents of canine lobular orbital adenomas then further investigation of papillomavirus vaccines may be warranted.

Viral pathogens have not been evaluated as a causative agent in cases of canine lobular orbital adenomas. Therefore, the purpose of this study was to evaluate conjunctival and orbital tissue from phenotypically normal dogs and from canine lobular orbital adenoma tissue samples for the presence of papillomavirus DNA. We hypothesized that papillomavirus DNA would not be detected in tissue from phenotypically normal dogs and that papillomavirus DNA would be detected in canine lobular orbital adenoma tissue samples.

## Results

All dogs in the phenotypically normal control group were mixed breed dogs and estimated to be between 2 and 9 years of age. All dogs were euthanized due to behavioral issues. No anterior segment changes were present in any of the dogs in this group. In addition, all normal control dogs were free of peri-ocular papillomas.

**Fig. 1:**
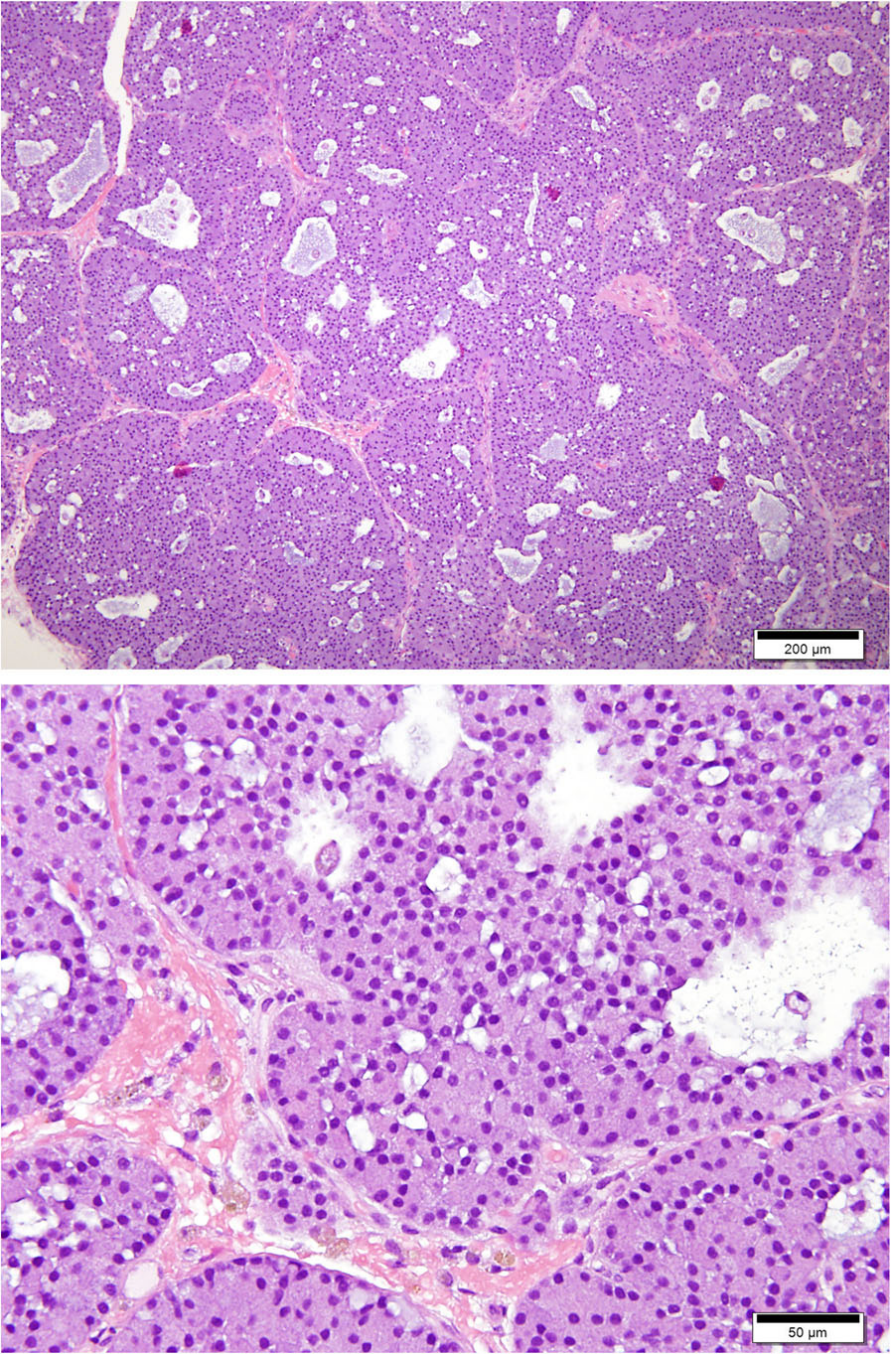
Canine lobular orbital adenoma. Photomicrograph illustrating well-differentiated glandular tissue lacking ducts. The epithelial cells are large and contain abundant cytoplasm and the nuclei are small and round. H&E 100x (top image); 400x (bottom image). *Photo courtesy of Dr. Megan Climans and the Comparative Ocular Laboratory of Wisconsin*

All formalin-fixed paraffin-embedded (FFPE) tissue samples from the canine lobular orbital adenoma group were confirmed canine lobular orbital adenomas via histopathology by the comparative ocular pathology lab of Wisconsin (COPLOW) utilizing the criteria described by Headrick et al. [1]. The histopathology of one of the tumors is shown in Fig. 1.

**Fig. 2:**
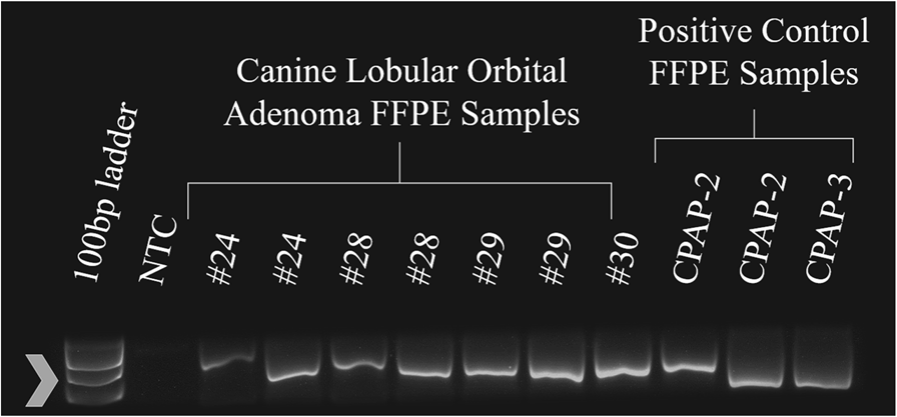
PCR for canine GAPDH sequence from canine lobular orbital adenoma FFPE samples. Amplicons were detected in all samples. The arrowhead indicates the position of the 400 base pair amplicon expected for this assay. NTC – Non-template control; CPAP – Canine papillomavirus positive control samples

**Fig. 3:**
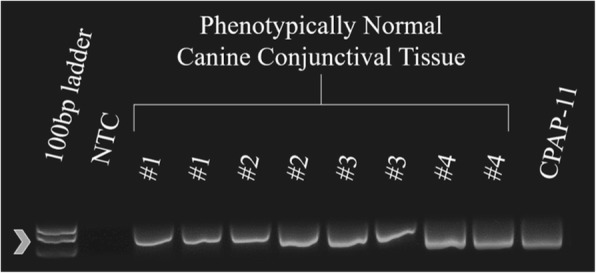
PCR for GAPDH sequence from freshly collected phenotypically normal canine conjunctival tissue samples. Amplicons were detected in all samples. The arrowhead indicates the position of the 400 base pair amplicon expected for this assay. NTC - Non-template control; CPAP – Canine papillomavirus positive control samples

**Fig. 4:**
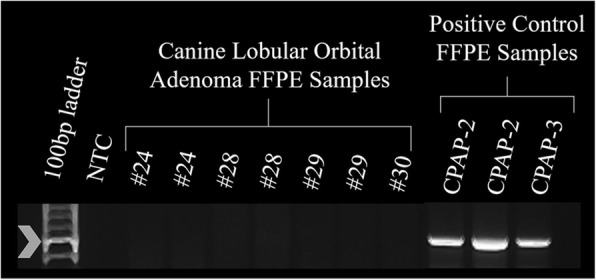
PCR for papillomaviral sequence from canine lobular orbital adenoma FFPE samples. Amplicons were detected in the positive control samples. No amplicons were detected in any of the FFPE orbital adenoma samples. The arrowhead indicates the position of the 480 base pair amplicon expected for this assay. NTC – Non-template control; CPAP – Canine papillomavirus positive control samples

**Fig. 5:**
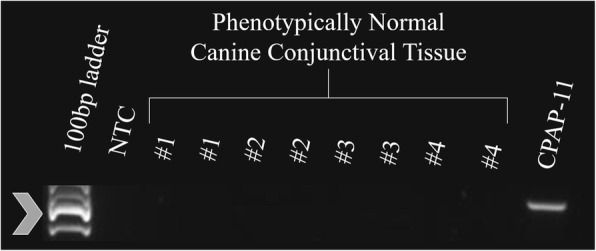
PCR for papillomaviral sequence from freshly collected phenotypically normal canine conjunctival tissue samples. Amplicons were detected in the positive control samples. No amplicons were detected in any of the normal conjunctival samples. The arrowhead indicates the position of the 480 base pair amplicon expected for this assay. NTC - Non-template control; CPAP – Canine papillomavirus positive control samples

The average age of dogs with lobular orbital adenomas was 10.5 years (range 6–16 years). The left eye was affected in 17 cases, the right eye in 11 cases, and both eyes in 4 cases; one case was presumed to be OU based on follow-up; however, histopathology was not available for the second eye. The affected eye, breed, age, and gender of each dog is provided in Table 1. DNA quantity and purity were deemed sufficient for PCR after evaluation by Qubit and Nanodrop. Amplification of an appropriate-sized band for GAPDH, a housekeeping gene, was detected in all samples, confirming that DNA extraction yielded DNA of sufficient quantity and quality for PCR (Figs. 2 and 3) [32–34]. Polymerase chain reaction results yielded no bands in the non-template negative controls and appropriate bands in the positive controls (three canine papillomas and one equine sarcoid). Papillomavirus DNA was not detected in fresh conjunctival and orbital tissue of phenotypically normal dogs. Also, papillomavirus DNA was not detected in samples of fresh or FFPE canine lobular orbital adenoma tissue (Figs. 4 and 5).

**Table 1:** Signalment of each canine lobular orbital adenoma dog included in the study, including affected eye, breed, age, and gender

	Affected Eye	Breed	Age (years)	Gender	Neuter Status
1	OS	Golden Retriever	12	Male	Intact
2	OD	Shepherd mix	10	Male	Neutered
3	OS	German Shepherd mix	8	Female	Spayed
4	OS	Mix	10	Female	Spayed
5	OD	Samoyed	11	Male	Neutered
6	OS	Labrador Retriever	11	Male	Neutered
7	OS	Boston Terrier	8	Female	Spayed
8	OD	Labrador Retriever	14	Female	Spayed
9	OS	Bichon Frise	12	Female	Spayed
10	OD	Labrador Retriever	7	Female	Spayed
11	OS	Labrador Retriever	8.3	Male	Neutered
12	OD	Dalmatian	–	–	–
13	OD	Australian Cattle Dog	6	Female	Spayed
14	OS	Samoyed	11	Female	Spayed
15	OS	Shih Tzu	9	Female	Spayed
16	OD	Mix	16	Male	Neutered
17	OS	Shih Tzu	10	Female	Spayed
18	OS	Cockapoo	8.5	Female	Spayed
19	OS	Labrador Retriever	12	Female	Spayed
20	OS	Labrador Retriever	–	Male	Neutered
21	OS	Samoyed	10	Female	Spayed
22	OD	Toy Poodle	12	Male	Neutered
23	OD	Wheaten Terrier	–	Male	Neutered
24	OD	Boston Terrier	13	Female	Spayed
25	OD	Samoyed	13	Female	Spayed
26	OS	Labrador Retriever	11	Female	Spayed
27	OS	Labrador Retriever	8	Female	Spayed
28	OD	Shih Tzu	12	Female	Intact
	OS				
30	OD	Miniature Poodle mix	9	Male	Neutered
	OS				
32	OD	Mix	10	Female	Spayed
33	OU (OD)	Golden Doodle	10	Male	Neutered
34	OS	Cockapoo	13	Female	Spayed
35	OS	Mix	11	Female	Spayed
	OD				

Data unavailable, *OS* oculus sinister (left eye), *OD* oculus dextrus (right eye), *OU* oculus uterque (each eye) Data unavailable, *OS* oculus sinister (left eye), *OD* oculus dextrus (right eye), *OU* oculus uterque (each eye)

## Discussion

This study is the first to evaluate papillomavirus’ role in canine lobular orbital adenoma. Papillomavirus DNA was not detected in any tissue from phenotypically normal dogs or dogs with canine lobular orbital adenomas. An association between papillomavirus and the development of canine lobular orbital adenomas is unlikely.

Tumors of the lacrimal and salivary glands are rarely reported in the veterinary literature, but include benign adenomas, malignant adenocarcinomas, and tumors of mixed epithelial and mesenchymal origin [35, 36]. Canine lobular orbital adenomas are rare benign orbital tumors in dogs with suspected lacrimal gland origin; however, zygomatic salivary gland or gland of the third eyelid cannot be excluded as the gland of origin in these cases [1]. These tumors can have devastating ocular consequences due to difficulty obtaining complete excision leading to subsequent recurrence. To date, an underlying cause for these tumors has not been determined.

There are a number of benign tumors of lacrimal and salivary gland origin. While the role of papillomavirus in human salivary gland tumorigenesis is controversial, HPV, including the more pathogenic strains HPV 16 and 18, has been identified in benign human salivary gland tumors [7, 8, 37]. Several studies have detected HPV in benign tumors of salivary gland origin, including pleomorphic adenomas, Warthin’s tumors, [8–10, 38] and salivary gland oncocytomas [37]. Thus, it is reasonable to evaluate benign glandular tumors in dogs for the presence of canine papillomavirus.

Several other studies have evaluated normal and abnormal canine ocular tissue for the presence of papillomavirus. Papillomavirus has been detected in conjunctival plaques and viral papillomas, and an eyelid squamous cell carcinoma in dogs [23, 28, 39]. However, Beckwith-Cohen et al. showed that papillomavirus was not present in 10 conjunctival squamous papillomas or normal conjunctival tissue from one dog [39]. Our current study is consistent with the Beckwith-Cohen study as papillomavirus was not present in normal conjunctival tissue or tissue from canine lobular orbital adenomas.

There are several explanations that support the lack of detection of papillomavirus DNA in our samples. The most likely is that papillomavirus is not a normal resident of canine conjunctival tissue and is not associated with the development of these tumors and therefore, papillomavirus DNA was not detected. However, fixation in formalin can lead to DNA fragmentation, [40] which may have impaired DNA amplification and led to false negative results in the present study. However, this is unlikely as several studies have detected papillomavirus DNA, including canine papillomavirus 12 and a novel virus closely related to CPV 16, via PCR with the FAP59 and FAP64 primers using FFPE tissue [41–43]. Additionally, three of the positive control samples were from FFPE tissue and this tissue was collected and processed in the same manner as the lobular adenoma tissue, suggesting that DNA fragmentation as a cause of the negative results is not likely to have occurred in this study. Another explanation is that papillomavirus levels were too low for detection and thus not detected. It is also possible that the PCR primers used in this study failed to detect papillomavirus DNA as these primers amplify the gene of the L1 capsid which is conserved among most, but not all papillomavirus families. These primers have been shown to amplify canine papillomaviruses 1–5 and 7 [44]. Further, previous studies have shown that viral DNA may lead to initial transformation of cells and tumor development, but are not required for maintenance of altered cells [45]. Thus it is possible that papillomavirus DNA was present to initiate tumor development, but was then lost resulting in negative PCR results. However, this phenomena is theorized to occur more commonly in malignant tumors and since canine lobular orbital adenomas are benign, this is unlikely to be the case [46–49]. Finally, another virus besides papillomavirus may be associated with development of canine lobular orbital adenomas. Further investigation of alternative infectious or genetic etiologies is warranted.

Limitations of this study include the relatively small sample size of both the orbital adenoma and phenotypically normal dogs. Further, affected FFPE tissue was compared to freshly collected tissue from normal dogs instead of FFPE tissue. While some may argue that a better comparison would have been to compare the canine lobular orbital adenoma tissue to age, breed, and sex matched FFPE conjunctival tissue sample, the authors elected to use fresh tissue from phenotypically normal dogs as this approach yields the highest quality DNA for amplification. As discussed earlier, formalin fixation and paraffin embedding can lead to cross-linking and fragmentation of the DNA. Therefore, fresh tissue from dogs euthanized at local humane shelters was used to eliminate the possibility of altering the DNA and to achieve the best representation of DNA from conjunctival tissue.

## Conclusions

In conclusion, papillomavirus DNA was not detected in fixed or fresh tissue samples of canine lobular orbital adenoma tumors and an association between papillomavirus and canine lobular orbital adenoma development is unlikely. Further research is needed to evaluate the etiology and pathogenesis of canine lobular orbital adenomas.

## Methods

### Phenotypically normal control group

Ten dogs, euthanized at local animal shelters for reasons unrelated to this study, were identified for use as phenotypically normal controls. All dogs underwent an ophthalmic examination of the anterior segment and adnexa prior to euthanasia and tissue was collected if dogs were free of gross ocular pathology. Excisional biopsies of conjunctival and orbital tissues were aseptically obtained from the superior and inferior conjunctival fornix of each eye immediately following euthanasia. After collection, fresh tissue was flash frozen in liquid nitrogen and stored at − 80 °C until DNA extraction.

### Canine lobular orbital adenoma group

Thirty six formalin-fixed paraffin-embedded (FFPE) tissue samples from 33 dogs previously diagnosed with canine lobular orbital adenoma were obtained from the Comparative Ocular Pathology Lab of Wisconsin (COPLOW). The histopathologic criteria outlined by Headrick et al. was used to confirm the diagnosis of canine lobular orbital adenoma for each tissue sample [1]. In addition, a fresh tumor sample, previously diagnosed by COPLOW as a canine lobular orbital adenoma, was collected for analysis, flash frozen in liquid nitrogen and stored at − 80 °C until DNA extraction.

### DNA extraction and polymerase chain reaction

Each FFPE tissue block was aseptically sectioned into 10 μm tissue scrolls. Genomic DNA was extracted from all samples, using the commercially available Qiagen DNeasy Blood and Tissue DNA Extraction Kit (Qiagen, N.V., Hilden, Germany) for the fresh tissue samples and the commercially available Qiagen QIAmp DNA FFPE Tissue Kit (Qiagen, N.V., Hilden, Germany) for the FFPE samples. The genomic DNA was quantified using the Qubit 2.0 fluorometer (Invitrogen, Life Technologies, Carlsbad, CA, USA) following the manufacturer’s protocol for the Quant-iT dsDNA HS Assay. Additionally, DNA quantity and purity were assessed using the NanoDrop 2000 Spectrophotometer (Thermo Fisher Scientific, Wilmington, DE, USA) following the manufacturer’s protocol. The purity of each DNA sample was determined by measuring the absorbance ratio at 260/280 nm. DNA was stored at − 20 °C until PCR was performed.

A specific 480 base pair fragment of the well-conserved papillomavirus L1 capsid protein was amplified by PCR using a set of degenerate primers, FAP59 and FAP64 (Table 2) [41]. This PCR primer set has been used extensively to sensitively detect papillomaviruses from a variety of animals including feline, equine, and canine neoplastic tissue samples [22, 29, 42, 50–52]. Non-template samples served as negative controls. Three FFPE tissue samples (two from canine viral papillomas and one from an equine sarcoid) and one fresh canine viral papilloma served as positive controls. Each PCR mixture contained 50 ng of DNA template for the FFPE samples and 5 ng of DNA template for the fresh tissue samples, 1.25 μL of the FAP59 and FAP64 primers (concentration 10 μM), 1.5 mM MgCl_2_, 0.2 mM each dNTP (New England BioLabs, Ipswich, MA, USA), 1.25 U *Taq* DNA polymerase (Invitrogen, Life Technologies, Carlsbad, CA, USA), and appropriate volumes of nuclease free water to reach a total volume of 50 μL for each reaction. All PCR reactions were performed in duplicate in an automated thermocycler (C1000 Thermal Cycler, Bio-Rad Laboratories, Hercules, CA, USA), using the following parameters: 10 min at 94 °C and then 45 cycles of 90 s at 94 °C, 90 s at 50 °C, 90 s at 72 °C, and finally 5 min at 72 °C. Polymerase chain reaction products were combined with Gel Red (GelRed® Nucleic Acid Gel Stain, Biotium, Inc., Fremont, CA, USA), electrophoresed in 1.5% agarose gel, and imaged in UV light to determine the presence of the specific amplicon band.

**Table 2:** Description of papillomavirus DNA primers, FAP59 and FAP64. Sequences of FAP59 and FAP64 degenerate primers

Primer	Polarity	Sequence	Expected amplicon length
FAP59	Forward	5′ TAACWGTIGGICAYCCWTATT 3′	480 bp
FAP64	Reverse	5′ CCWATATCWVHCATITCICCATC 3′	

Degenerate nucleotides: W = T, C; I = inosine; Y = C, T; H = A, C, T; V = A, C, G

To confirm the presence of DNA in each sample, an approximate 400 base pair segment of the glyceraldehyde-3-phosphate dehydrogenase (GADPH) gene was amplified using canine GAPDH primers as previously described [32–34]. Each PCR mixture contained 50 ng of DNA template, 1.0 μL each of the forward and reverse primers (concentration 10 μM), 2.5 μM MgCl_2_, 0.2 mM each dNTP (New England BioLabs, Ipswich, MA, USA), 2.5 U *Taq* DNA polymerase (Invitrogen, Life Technologies, Carlsbad, CA, USA), and appropriate volumes of nuclease free water to reach a total volume of 25 μL for each reaction. Polymerase chain reaction was performed in the aforementioned automated thermocycler using the following parameters: 5 min at 94 °C and then 32 cycles of 60 s at 94 °C, 60 s at 58 °C, 60 s at 72 °C, and finally 5 min at 72 °C. Polymerase chain reaction products were combined with Gel Red (GelRed® Nucleic Acid Gel Stain, Biotium, Inc., Fremont, CA, USA), electrophoresed in 2% agarose gel, and imaged in UV light to determine the presence of the specific amplicon.

## Data Availability

The datasets used during the current study are available from the corresponding author on reasonable request.

## Abbreviations

COPLOW: Comparative Ocular Pathology Lab of Wisconsin
FFPE: Formalin-fixed paraffin-embedded
HPV: Human Papillomavirus
OD: Oculus dextrus (right eye)
OS: Oculus sinister (left eye)
OU: Oculus uterque (each eye)
PCR: Polymerase chain reaction
